# Senescent AECⅡ and the implication for idiopathic pulmonary fibrosis treatment

**DOI:** 10.3389/fphar.2022.1059434

**Published:** 2022-11-15

**Authors:** Tingwei Zhang, Jinjin Zhang, Changjun Lv, Hongbo Li, Xiaodong Song

**Affiliations:** ^1^ Department of Respiratory and Critical Care Medicine, Binzhou Medical University Hospital, Binzhou Medical University, Binzhou, China; ^2^ Department of Cellular and Genetic Medicine, School of Pharmaceutical Sciences, Binzhou Medical University, Yantai, China

**Keywords:** alveolar type II epithelial cells (AECIIs), cell senescence, idiopathic pulmonary fibrosis, senescence-associated secretory phenotype, senescenceassociated differentiation disorder

## Abstract

Idiopathic pulmonary fibrosis (IPF) is a chronic and lethal lung disease with limited treatment options. The onset of IPF increases with age, indicating that aging is a major risk factor for IPF. Among the hallmarks of aging, cellular senescence is the primordial driver and primary etiological factor for tissue and organ aging, and an independent risk factor for the progression of IPF. In this review, we focus on the senescence of alveolar type II epithelial cells (AECIIs) and systematically summarize abnormal changes in signal pathways and biological process and implications of senescent AECIIs during IPF progression. Meanwhile, we objectively analyze current medications targeting the elimination of senescent cells or restoration of vitality such as senolytics, senomorphics, autophagy regulators, and stem cell therapy. Finally, we dialectically discuss the feasibility and limitation of targeting senescent AECIIs for IPF treatment. We hope that the understanding will provide new insights to the development of senescent AECII-based approaches for the prevention and mitigation of IPF.

## Introduction

Idiopathic pulmonary fibrosis (IPF) is an irreversible fibrotic disease in the lungs and is the most common form of idiopathic interstitial pneumonia and idiopathic fibrotic lung disorder (Moss et al., 2022). Its biological process is defined as an abnormal repair response to repeated alveolar epithelial cells (AEC) damage and fibroblast-to-myofibroblast differentiation and characterized by the excessive disordered deposition of collagen in the extra- and intra-cellular matrix (Liu and Liu, 2020). Several potential risk factors, such as aging, genetic predisposition, chemical, environmental exposure, and bioenvironmental factor (bacteria and virus), can act on various types of lung cells and enhance the risk of developing IPF (Noble et al., 2012; Moore and Moore, 2015; Sheng et al., 2020; Parimon et al., 2021). Of these risk factors, aging is considered an independent risk factor. Even in patients with a genetic predisposition, the onset of IPF seldom occurs before the sixth decade, and the incidence increases exponentially with advancing age (Raghu et al., 2006). A longitudinal cohort study identifying independent risk factors for the progression of interstitial lung disease has shown that the risk of IPF in people aged 70 or over is 6.9 times that in people aged over 40 (Choi et al., 2018), confirming that IPF is an age-related disease.

Cell senescence and stem cell exhaustion are the hallmarks of all age-related diseases, as in IPF. Alveolar type II epithelial cells (AECIIs) are the stem cells for the lungs and play a role in maintaining intrapulmonary homeostasis, immunity, and regeneration in the alveoli. Senescent AECIIs secrete high levels of interleukin, interferon, tumor necrosis factor, colony-stimulating factors, growth factors, and chemotactic cytokines, which promote fibroblast-to-myofibroblast differentiation and persistent tissue remodeling (Abbadie et al., 2017; Liu and Liu, 2020). A recent study has uncovered that pulmonary fibrosis after coronavirus disease 2019 (COVID-19) may be caused by virus-induced AECII senescence (Lee et al., 2021; Michalski et al., 2022; Sinha et al., 2022). Preventing AECII senescence or targeting senescent cells in patients with COVID-19 can reduce the risk of pulmonary fibrosis (Hong et al., 2022). Moimas et al. detected that AECII exhibited high levels of the senescence markers p21 and p16 from patients with IPF (Moimas et al., 2019). Other numerous studies have shown that AECII senescence promotes the occurrence of IPF (Naikawadi et al., 2016; Parimon et al., 2020; Yao et al., 2021; Hong et al., 2022). However, pathological mechanisms underlying AECII senescence and specific effects of targeting senescent AECIIs on IPF remain unclear. This review will discuss the mechanism of AECII senescence, which drives the onset and progression of IPF, and highlights the advantages and disadvantages of targeting senescent AECIIs for IPF.

## Cellular senescence is a risk factor for idiopathic pulmonary fibrosis

### Cellular senescence

Cellular senescence is irreversible cell cycle arrest, characterized by morphological flattening and expansion, resistance to apoptosis, altered gene expression and chromatin structure, expression of senescence-associated β-galactosidase (SA-β-gal) and acquisition of a senescence-associated secretory phenotype (SASP) (Mohamad et al., 2020). Often caused by persistent DNA damage. It is worth mentioning that during cellular senescence, an increase in SA-β-gal was exhibited, which was the first marker for *in situ* detection of senescence cellular in tissues (Kurz et al., 2000). Cellular senescence on an organism has three adverse effects: First, the degenerative physiological function of senescent cells and their accumulation disrupt tissue and organ functions, thereby leading to the aging of the body. Second, accumulated senescent cells secrete a variety of proteins such as inflammatory cytokines, chemokines, growth factors and matrix metalloproteinases. This phenomenon is known as the senescence-associated secretory phenotype (SASP), SASP affects the physiological functions of normal cells in the surrounding microenvironment in an autocrine or paracrine manner, causing the dysfunction of tissues and organs and participating in senescence-associated diseases (Childs et al., 2015). Third, stem cells are gradually exhausted during the aging process. When tissues and organs function abnormally, stem cells cannot be repaired in time, thus affecting the physiological functions of the body (Korolchuk et al., 2017; Parimon et al., 2021). Accompanied by the adverse effects of cellular senescence on an organism, abnormal changes arise in biological processes, such as telomere attrition, DNA damage, epigenetic modifications, abnormal protein homeostasis, mitochondrial dysfunction, and impaired autophagy (Korfei et al., 2020; Duckworth et al., 2021; Kellogg et al., 2021).

The regulatory pathways of cellular senescence mainly include cell cycle and SASP regulation (Parimon et al., 2021). Cell cycle blockade is induced by cyclin-dependent kinase (CDK) inhibitors p16^Ink4a^ or p53/p21^Cip1/Waf1^ (Lomas et al., 2012). Persistent DNA damage induces the expression of p16 through the transcription factor Ets and causes the stabilization of transcription factor p53, which induces the expression of p21. P16 and P21 respectively inhibit the cyclin cyclin-dependent kinase 4/6 (CDK4/6) complex and cyclin-dependent kinase 2 (CDK2). Both CDK inhibitors activate the Rb protein, which blocks the cell cycle and leads to cellular senescence (Ohtani et al., 2001; Takahashi et al., 2006; Yasuda et al., 2021). Therefore, p16^INK4a^ and p21^WAF1^ are considered to be important markers of cellular senescence. However, no specific marker for cellular senescence has been identified. The study shown that senescent cells synthesize many oxidized lipids, a class of bioactive lipids derived from the oxidation of polyunsaturated fatty acids. Dihomo-15-deoxy-delta-12,14-prostaglandin J2 (dihomo-15d-PGJ2) as an oxygenated lipid that promotes senescence and secretion of SASP by activating RAS/mitogen activated protein kinase (RAS/MAPK) pathway. It is produced and accumulated in senescent cells, released upon senescent cell lysis, and detected in urine and blood. Wiley et al. (2021) confirmed dihomo-15d-PGJ2 as a potential biomarker for validating the performance of aging drugs. Therefore, it may become the first biomarker of aging, or even a therapeutic target in the near future. Several studies have shown that cellular senescence mediates IPF, targeting senescence can alleviate fibrosis and extend the life spans of experimental animals (Baker et al., 2016; Schafer et al., 2017; Hohmann et al., 2019; Rhinn et al., 2019; Omori et al., 2020). However, little is known about the mechanisms by which senescence leads to IPF.

### Cellular senescence in idiopathic pulmonary fibrosis

The human lung, the organ with the largest surface area in the body, represents a unique interface with the external environment. Lung maturation and function peak around 25 years of age, remain steady until 35 years of age, and gradually decline with structural remodeling thereafter (Ruaro et al., 2021; Schneider et al., 2021), which characterized by enlarged alveolar size and signifies increased mechanical force (Schiller et al., 2019). The progressive increase in alveolar size as the lungs senescence partially explains why the elderly are more likely to suffer from IPF in terms of mechanical force. In recent years, developments in mechanics and mechanical force, including mechanical stiffness, stretch, adhesion, density and tension, have been thought to be the key factors in IPF remodeling (Chilosi et al., 2013). They not only induce cellular senescence but also promote AECII proliferation, differentiation, and alveolar regeneration by regulating multiple signaling pathways (Wu et al., 2021). Elevated mechanical tension can activate TGF-β signaling loop in AECIIs, which drives the periphery-to-center progression of lung fibrosis (Gonzalez-Garcia et al., 2018; Wu et al., 2021). Transforming growth factor *β* (TGF-β) is a multifunctional cytokine that controls growth, proliferation, differentiation and apoptosis in many cell types. It includes TGF-β isoforms (TGF-β1, TGF-β2 and TGF-β3) and other signaling proteins (Meng et al., 2016; Morikawa et al., 2016). Numerous studies have demonstrated a significant increase in TGF-β1 gene and protein expression in fibrotic lung tissue (Bellaye et al., 2018; Boutanquoi et al., 2020; Lee et al., 2020; Lv et al., 2020). In IPF, TGF-β leads to increased ECM production mainly through induction of fibroblast activation and myofibroblast differentiation (Liu et al., 2021). Circular RNA (circRNA) is found to be associated with pulmonary fibrosis (Zhou et al., 2022). It produces from pre-mRNA back splicing, is a kind of endogenous noncoding RNA. A total of 67 significantly dysregulated circRNAs were identified in the plasma of IPF patients by using a circRNA microarray, including 38 upregulated-circRNAs and 29 downregulated-circRNAs (Li et al., 2018; Li et al., 2019). Among these circRNAs, the upregulated has-circ-100906, termed circANKRD42 because it is derived from its host gene Ankyrin repeat domain 42 (ANKRD42), increases with age in healthy people. It can accelerate IPF by mediating the crosstalk between mechanical stiffness and biochemical signal (Xu et al., 2022). Mechanistic studies reveal that circANKRD42 sponges miR-324-5p and miR-136-5p to promote the expression of yes-associated protein 1 (YAP1). Accumulating YAP1 in nucleus bound to tea domain transcription factor (TEAD), which initiates the transcription of genes related to mechanical stiffness.

In IPF, cellular senescence can lead to stem cell failure, SASP secretion, impaired myofibroblast apoptosis (Liu and Liu, 2020), and senescence-associated differentiation disorder (SADD), manifested by reduced tissue regeneration and excessive cellular matrix deposition. Impaired myofibroblast apoptosis, excessive deposition at an injury site, and continuous response are also considered the base for the continuous IPF process (Liu and Liu, 2020; Wei et al., 2021). Studies confirmed that targeting senescence cells is effective in alleviating IPF and senescence-related diseases (Lehmann et al., 2017; Merkt et al., 2020). Therefore, further exploring cellular senescence mechanisms and the involvement of senescent cells in the pathogenesis of IPF can facilitate the discovery of novel therapies for IPF treatment and other senescence-related diseases.

## Senescent AECIIs promote idiopathic pulmonary fibrosis development

### Role of AECIIs

AECs are composed of alveolar type I epithelial cells (AECIs) and AECIIs. They are separated from one another by interalveolar septa. AECIs are differentiated cells with a thin and flat shape and cover over 90% of the alveolar surface area, facilitating contact among alveolar endothelial cells and promoting gas exchange. AECIIs are small and cuboidal cells covering the remaining 10% of the alveolar surface area. Their primary function is to reduce surface tension by secreting surfactants (Confalonieri et al., 2022). When lung tissues are damaged, AECIIs can differentiate into AECIs, which are involved in epithelial repair (Barkauskas et al., 2013). In recurrent micro-injuries, dysfunctional AECIIs not only fail to maintain physiological lung regeneration but also promote abnormal epithelial–mesenchymal crosstalk, which lead to fibrosis rather than regeneration (Confalonieri et al., 2022). Mature AECIIs have heterogeneity, telomerase activity, and proliferative and differentiation potential. They respond to various cellular signals during senescence (Chen et al., 2017), and are among the major effector cells in the evolution of IPF. Therefore, understanding the mechanisms involved in the involvement of AECII as stem cells in lung repair is of great importance in delaying or treating pulmonary fibrosis. Their quiescence, proliferation, and differentiation are regulated by a combination of signaling pathways, including Notch, Hippo/Yap, and TGF-β1 pathways (Aumiller et al., 2013; Wu and Song, 2020).

The Hippo/YAP kinase cascade reaction promotes alveolar regeneration induced by proliferation of lung parenchymal cells. It comprises a large protein network, whose central components can be divided into two modules: the regulatory kinase module in the cytoplasm and the transcription module in the nucleus (Sun et al., 2021). The kinase module mainly comprises mammalian Ste20-like serine/threonine protein kinase 1 and 2 (MST1/2) and the large tumor suppressor kinase 1 and 2 (LATS1/2) axis (Moya and Halder, 2019). They interact with the scaffolding protein Salvador (SAV1) and ultimately phosphorylate Yap and its homologue TAZ in the transcriptional module to prevent nuclear localization. Most importantly, activation of the upstream kinase module hinders the downstream transcription module (Sun et al., 2021). When in the absence of this phosphorylation cascade, YAP/TAZ localizes to the nucleus to regulate gene transcription associated with cell migration and proliferation. Nuclear translocation of YAP/TAZ can promote AECII differentiation into AECI. Studies shown that non-cell-specific YAP/TAZ RNA interference can exacerbate bleomycin-induced pulmonary fibrosis in mice by blocking alveolar regeneration (Haak et al., 2019).

There have been conflicting reports on the relationship between TGF-β signaling and alveolar epithelial cells. For example, when IL-33 was transferred into Treg-depleted mice during the study of ARDS, Treg cell recovery and a significant increase in TGF-β1 secretion were observed, and lung regeneration was promoted by accelerating the recovery of AECII, presumably IL-33-dependent Tregs accumulation may accelerate lung epithelial regeneration in a TGF-β1-dependent manner (Tan et al., 2021). This validates that TGF-β1 promotes regeneration of alveolar epithelial cells. In addition, single-cell RNA sequencing also identified TGF-β signaling as a key factor in lipopolysaccharide (LPS)-induced regeneration after lung injury in mice (Riemondy et al., 2019). However, it has also been demonstrated that TGF-β1 promotes lung fibrosis by inducing apoptosis in AECII. Recent studies have found that in bleomycin-induced lung fibrosis, the early production of TGF-β and platelet derived growth factor subunit A (PDGFA) by senescent AECII may directly promote fibroblast activation and collagen production, which in turn promotes fibrosis (Yamada et al., 2022). In addition to the above signaling pathways, growth factors produced by different neighboring cells, intercellular contacts, immune cells, and the extracellular matrix also regulate the state of AECIIs (Wu and Song, 2020).

### Pro-fibrotic role of senescent AECII

AECII senescence promotes IPF (Parimon et al., 2020), and the senescent phenotypes of AECIIs are detected in fibrotic areas in the lungs of patients with IPF. These features confirm that senescent AECIIs have capacities for incomplete repair and can promote fibroproliferation (Wiley et al., 2021). To study the role of AECII senescence in IPF, Yao et al. (2021) established a mouse model with a conditional deletion of Sin3a in AECIIs and found that conditionally induced AECII senescence leads to progressive fibrosis and removing senescent AECIIs from the lungs of Sin3a LOF mice prevents progressive fibrosis, suggesting that targeting senescent AECIIs can prevent progressive fibrosis. Moreover, a study has confirmed that targeting senescent AECIIs can stabilize epithelial cell phenotype and reduce fibrosis markers (Lehmann et al., 2017).

Telomere is a ribonucleoprotein complex at the end of a cell’s chromosome, consisting of an oligonucleotide sequence and a corresponding protein, which is essential for chromosome stability (Rossiello et al., 2022). Telomere is highly susceptible to various stresses. Cellular stress signals trigger glycogen synthase kinase-3β targeting the telomere protection protein complex, inducing the telomerase recruitment protein tripeptidyl peptidase I (TPP1) phosphorylation, and promoting TPP1 multisite polyubiquitination and degradation, resulting in telomere uncapping, which in turn activates protein kinase inhibitors (Razdan et al., 2018; Hong et al., 2022). H_2_O_2_-induced AECII model, bleomycin-treated mice and IPF patients samples show that AECIIs exhibit abnormal shortening of telomeres. In addition to this, incomplete replication of chromosome ends can lead to telomere shortening (Pineiro-Hermida et al., 2022). Recently, Pineiro-Hermida et al. (2022) studied the telomeres of different cell types in the lung by controlling the TRF1 gene and confirmed that fibrosis only occurs when telomeres are disrupted in AECII, and telomere shortening can be compensated by the telomerase reverse transcriptase (TERT) and telomerase RNA component (TERC) (Pineiro-Hermida et al., 2022). Thus, preventing stress-induced telomere damage may prevent cells from entering replicative senescence and fibrosis. Targeting senescent AECII or using inhibitors of telomere dysfunction to prevent senescence is a promising strategy for IPF intervention (Wang et al., 2020; Hong et al., 2022). Peptidomimetic telomere dysfunction inhibitor TELODIN, a newly discovered 8-mer peptide with TPP1 protective function that can reduce telomere uncapping and shortening, thereby expanding the alveolar AECII stem cell population in mice and preventing chronic stress-induced premature lung senescence and fibrosis, thus serving as a novel tool for disrupting pulmonary fibrosis (Wang et al., 2021). Long noncoding RNA (lncRNA) is usually unable to code protein and has a length more than 200 nt, which can inhibit or promote lung fibrogenesis and is becoming a promising new target (Song et al., 2014a; Zhang B et al., 2021). lncRNA telomeric repeat-containing RNA (lncTERRA) is a physiological indicator of aging for IPF. Gao et al. (2017) found that RNA interference on lncTERRA can ameliorate the functions of telomeres, thereby alleviating the symptoms of pulmonary fibrosis such as forced vital capacity.

As AECIIs become senescent, their stem cell potential is gradually exhausted. This effect not only cause impaired self-proliferation but also hamper the differentiation of AECIIs into AECIs, which leaves AECIIs in an intermediate or partially differentiated state, also known as the pre-alveolar type I transitional cell state or alveolar differentiation intermediate, resulting in SADD. When AECIIs are in a state of transition or partial differentiation, they can promote the proliferation of myofibroblasts in a low-inflammation environment created by SASP, which promote the development of IPF (Hong et al., 2022). Watanabe et al. (2021) has confirmed that the incomplete conversion of AECs from AECIIs into AECIs can lead to the development of pulmonary fibrosis. During the differentiation of AECⅡs into AECIs, AECⅡs undergo dramatic cell shape changes (Wu et al., 2021). When SADD occurs in senescent AECIIs, it not only promotes aberrant trans-differentiation but also leads to impaired AECⅡ regeneration during the repair of AECs. Impaired regeneration results in the exposure of AECIIs to continuously elevated mechanical force caused by cytoskeleton rearrangement (Wu et al., 2021). Thus, SADD should be used as an entry point for the detection of premature lung failure and the early stages of fibrosis. Single-AECII populations can be sequenced for the differentiation of the gene profile of AECII senescence and differentiation status, identification of structural modifications after gene transcription, and decoding of compromised protective complexes. An in-depth study of the mechanisms that promote lung regeneration and reduce elevated mechanical forces caused by impaired alveolar regeneration may improve interventions for IPF. The Notch signaling pathway plays an important role in the differentiation of AECII into AECI, it is activated in AECII early in alveolar repair but is inhibited by the Notch ligand deltalike 1 homolog (Dlk1) late in repair after Notch activation has reached its peak. Dlk1 controls Notch signaling after lung injury and promotes AECII to AECI conversion and alveolar epithelial repair. Lack of Dlk1 in AECII results in continued activation of Notch signaling in AECII, which prevents AECII from differentiating to AECI, resulting in incomplete alveolar repair. It suggesting that Dlk1 and the Notch signaling system may be potential therapeutic targets for alveolar epithelial repair (Liu et al., 2010; Finn et al., 2019). In-depth study of the Notch signaling pathway to facilitate the conversion of AECII to AECI, thus avoiding the development of SADD-induced IPF is a possible direction for future research.

### Senescent AECII and senescence-associated secretory phenotype

During AECII senescence, a specific phenotype is acquired, which is known as the SASP, which is characterized by replication arrest and the abnormal secretion of pro-fibrosis and pro-inflammatory senescence-related factors (Munoz-Espin and Serrano, 2014; Lehmann et al., 2017; Hansel et al., 2020). Proinflammatory and matrix-degradation factors are the most important ones (Coppe et al., 2008). SASP can lead to cellular dysfunction and impaired immune function and reacts on senescent cells and their neighbors, accelerating the aging process and thus creating a vicious cycle that maintains inflammation in the lungs. SASP acts as a trigger and effector molecule for the advancement of IPF (Rana et al., 2020). Studies have confirmed that KDM4, a key regulator of SASP, can selectively target SASP to inhibit AECII senescence while maintaining cell cycle arrest to manipulate senescent AECII, thereby maintaining homeostasis within tissues and organs and controlling organismal senescence (Zhang S et al., 2021). By contrast, the human protein 12S rRNA-c, encoded by mitochondrial DNA, and the mitochondrial open reading frame induce the production of specific SASP factors in azithromycin-induced and replicative cellular senescence (Kim et al., 2018). Therefore, targeting mitochondria to inhibit SASP may be a potential strategy for the treatment of IPF. Furthermore, the expression of noncoding RNAs is significantly up-regulated during cellular senescence. Further analysis has shown that human satellite II (hSATII) RNA interferes with the staining of certain SASP gene regions by impairing the function of CCCTC-binding factor. Pericentromeric hSATII RNA can promote SASP-Like inflammatory gene expression in senescent cells, thereby up-regulating the expression of SASP-like inflammatory genes (Miyata et al., 2021). In addition, studies have shown that telomere-mediated AECII senescence can lead to cell-autonomous defects and upregulation of secondary paracrine signaling, which can induce inflammation and mesenchymal abnormalities associated with altered intracellular gene expression and SASP (Alder et al., 2015). SASP affects its surrounding non-senescent cells through autocrine and paracrine functions and mediate the exacerbation of cellular senescence (De Cecco et al., 2019).

In IPF, SASP regulates senescence mainly through nuclear factor kappa-B (NF-κB), CCAAT-enhancer-binding protein beta (CEBPβ), and tumor protein p53 (TP53). All three of these transcription factors have a common characteristic which is their redox-dependent regulation. Among them, NF-κB is the main regulator of SASP, which is maintained in an autocrine manner by the SASP factor interleukin Iα (IL-Iα) (Nelson et al., 2012; Salminen et al., 2012). Secretion of IL-1α and signaling cascade of the p38 mitogen-activated protein kinase (p38MAPK) can active NF-κB, thereby promoting SASP expression (Orjalo et al., 2009; Amaya-Montoya et al., 2020). C/EBPβ mainly regulates the expression of various SASP factors. Its activation is a key event in the transition of cells to the terminal senescent state (Hoare et al., 2016). TP53 plays a key role in cellular stress response and senescence by regulating multiple antioxidant genes to maintain cellular redox homeostasis. It can promote p38MAPK phosphorylation and downstream NF-κB activation when p53 inactivated, thereby establishing an irreversible senescence phenotype (Freund et al., 2011). TGF-β is one of the secreted factors of SASP. When TGF-β signaling is activated in an autocrine or paracrine manner, it induces SASP and thus induces and maintains the aging phenotype. Interestingly, spectral tracing in mice has shown that AECIIs undergo aberrant cellular remodeling during increased TGF-β and TP53 signaling. This effect is accompanied by morphological changes associated with the replicative senescence characteristics of SADD (Yang et al., 2020).

Senescent AECIIs propagate senescence signals to surrounding cells by secreting SASP, which not only leads to persistent inflammation, tissue remodeling, and fibrotic phenotypic changes but also creates senescence-associated low-grade inflammation, leading to SADD that promotes myofibroblast proliferation under SALI (Acosta et al., 2013; Kobayashi et al., 2020) and further promoting IPF. In conclusion, a causal relationship among senescent AECⅡs, SASP, and SADD was found, which synergistically promotes the occurrence and progression of IPF. Therefore, preventing premature pulmonary failure or clearing senescent AECⅡs and blocking its related SASP secretion can be effective measures for IPF treatment.

### Senescent AECII and autophagy

Autophagy, a highly selective cellular clearance pathway associated with the maintenance of cellular tissue homeostasis, is an evolutionarily conserved degradation system in which cellular contents, such as proteins, organelles, and lipids are degraded in a lysosome-dependent manner (Kirkin, 2020). Autophagy inhibits senescence-associated inflammation, maintains genomic integrity, and preserves cellular and tissue homeostasis and the regenerative capacity of stem cells; the core processes are initiated by inhibiting mammalian target of rapamycin (mTOR) or activating Adenosine monophosphate (AMP)-activated protein kinase (AMPK) (Rubinsztein et al., 2011; Hansen et al., 2018). Studies show a decrease in AMPK phosphorylation and an increase in mTORC1 signaling and metabolic reprogramming in IPF (Hansen et al., 2018; Rangarajan et al., 2018). mTOR, a mammalian target of rapamycin, forms two complexes, mTORC1 and mTORC2. They have distinct effector proteins that are activated by multiple upstream inputs and trigger distinct downstream cellular responses (Plate et al., 2020). Among them, mTORC1 regulates autophagy and senescence by promoting *ab initio* lipid synthesis through sterol response element binding protein (SREBP), protein synthesis through phosphorylation of the eukaryotic translation initiation factor 4E-binding protein 1 (4E-BP1), and in the absence of glucose, the key energy sensor AMPK promotes autophagy through direct activation of unc-51-like kinase 1 (ULK1). Under nutrient-sufficient conditions, mTORC1 phosphorylates ULK1, preventing its activation and disrupting its interaction with AMPK to prevent autophagy (Kim et al., 2011).

Impaired autophagy is one of the hallmarks of senescence (Aman et al., 2021), and inflammation caused by its impairment is a major driver of senescence-induced tissue damage (Lopez-Otin et al., 2013; Franceschi et al., 2018). In addition, autophagy plays an important role in the control and treatment of COVID-19 (Brest et al., 2020; Mijaljica and Klionsky, 2020; Shojaei et al., 2020). The autophagic pathway is tightly regulated by multimolecular pathways, such as AMPK and mTORC1, and deacetylases (sirtuins) (Rubinsztein et al., 2011), and mTOR signaling is a central part of TGF-β1-mediated fibrosis (Laplante and Sabatini, 2012; Zhai et al., 2017). Severe deficiencies in autophagy levels in lung tissues during the IPF cause AEC senescence and damage, leading to abnormal epithelial–mesenchymal crosstalk and promoting fibroblast-to-myofibroblast differentiation. Meanwhile, restoring autophagy inhibits transformation and reduces collagen deposition, thereby inhibiting IPF formation (Araya et al., 2013; Margaritopoulos et al., 2013). Therefore, promoting autophagy to slow down senescence and regulating the IPF process through the disruption of typical molecular pathways is a promising therapeutic option. lncIAPF (lncRNA inhibit autophagy in pulmonary fibrogenesis) is identified as a profibrotic factor to promote pulmonary fibrosis. Mechanistically, lncIAPF forms a RNA-protein complex with human antigen R (HuR) to block autophagy by controlling the stability of the target genes EZH2 (enhancer of zeste 2 polycomb repressive complex 2 subunit), STAT1 (signal transducer and activators of transcription 1) and FOXK1 (forkhead box K1). Zhang et al. have provided preclinical evidence from the mouse models of lung fibrosis and patient samples and proposed pharmacological approaches for inhibiting lncIAPF related to autophagy; these approaches represent promising therapeutic options for IPF (Zhang et al., 2022). However, numerous questions need to be answered. For example, given that reduction in autophagy in IPF can lead to AEC senescence and the accumulation of inflammatory factors, and excessive autophagy can lead to lung atrophy, how autophagy balance can be achieved? What is the threshold for autophagy balance? How autophagy regulators can be delivered to specific cells at the right time? In the future, changes in AECII autophagy in IPF, the role and regulatory mechanism of autophagy in IPF, and whether AECII stem cell properties can be restored through autophagy for the conversion of AECIIs into AECIs and alleviation of SADD should be investigated.

## Medication

IPF is a serious lung disease with poor prognosis and without effective treatment. Anti-inflammatory drugs, corticosteroids and immunosuppressants have been used in the treatment of IPF over the past decades based on the assumption that inflammation is the main mechanism of pulmonary fibrosis (Hewlett et al., 2018). However, a multicentre randomised trial showed that the combination of corticosteroids, azathioprine and N-acetylcysteine increased mortality and hospitalisation rates (Raghu et al., 2012). As a result, this combination was discontinued. Only pirfenidone and nintedanib are currently approved for clinical use. In placebo-controlled, randomised phase 3 studies, pirfenidone and nitisinib effectively slowed disease progression, but did not improve survival or reverse pulmonary fibrosis (Lomas et al., 2012; King et al., 2014; Richeldi et al., 2014). Recent advances in understanding the pathogenesis of IPF are multifaceted and have led to the exploration and development of therapeutic agents for IPF. AECII, as stem cells of the alveolar epithelium, play an important role in the development of IPF due to the positive feedback loop caused by their senescence (Parimon et al., 2020; Kellogg et al., 2021; Parimon et al., 2021). Hashimoto et al. (2016) provided striking evidence that cellular senescence is a relevant target for lung functional improvement. Thus, one of the therapeutic strategies for IPF is to target senescent cells mainly through the elimination of senescent cells or restoration of their vitality. The following section will focus on three therapies targeting senescent AECIIs. Senotherapeutics, which is a new class of antisenescence agents designed to eliminate or delay the adverse effects of cellular senescence containing senolytics (selectively removes senescent cells) and senomorphics (acts as SASP inhibitors without killing cells) (Lagoumtzi and Chondrogianni, 2021), autophagy regulators, and stem cell therapy.

### Senolytics

Senolytics are agents that target senescent cells through senescence-associated anti-apoptotic pathways (Lagoumtzi and Chondrogianni, 2021). Kim et al. have summarized seven classes of antisenescence agents, namely, natural compounds, kinase inhibitors, Bcl-2 family inhibitors/Bcl-2 homolog 3 (BH3) mimetics, MDM2/p53 interaction inhibitors, Hsp90 inhibitors, p53 binding inhibitors, and HDAC inhibitors (Kim and Kim, 2019). The combination of quercetin and dasatinib (DQ therapy) can alleviate experimental pulmonary fibrosis by targeting senescent cells. The first human trial conducted on patients with IPF has validated the safety of DQ therapy (Justice et al., 2019). The AECII pathology-driven features in COVID-19 are similar to those in IPF (Sinha et al., 2022). Tests on mice infected with SARS-CoV-2 showed that DQ therapy or ABT-263 have targeted the elimination of virus-induced senescence and alleviation of COVID-19-related pulmonary symptoms. Further clinical trials conducted on COVID-19 patients have found that quercetin significantly improve pulmonary symptoms (Lee et al., 2021), but DQ therapy dose-dependently cleared senescent cells while damaging proliferating cells. The inhibition of Bcl-2/xl by ABT-263 can selectively target senescent AECIIs and reverses persistent pulmonary fibrosis in mice. However, it also results in the removal of normally proliferating or dormant cells from organs (off-target effect) (Pan et al., 2017). Research should further explore the key molecules and signaling pathways involved in AECII senescence causing IPF to preserve AECII population with differentiation potential in tissues and prevent the off-target effect. Damage to the stem cell characteristics of AECIIs in the microenvironment, which can lead to impaired differentiation and renewal and repair of tissues, should be prevented.

### Senomorphics

Senomorphics target SASP by inhibiting SASP-expression-related pathways or inhibit specific SASP factors and neutralizing specific antibodies. Senomorphics contain JAK inhibitors (ruxolitinib), NF-κB inhibitors (resveratrol and apigenin), mTOR kinase inhibitors (rapamycin and everolimus) and antibodies against specific SASP factor (Song et al., 2020). Metformin, ruxolitinib and glucocorticoids have been currently approved by the FDA as potential anti-aging drugs (Laberge et al., 2012; Lagoumtzi and Chondrogianni, 2021). Other reports have suggested that the Ca^2+^ channel blockers loperamide and niguldipine, the ataxia telangiectasia-mutated (ATM) kinase inhibitor KU-60019, and the IKK peptide inhibitor NBD have potential antisenescence activities (Kang et al., 2017; Song et al., 2020; Lagoumtzi and Chondrogianni, 2021). However, their targeting and efficacy have not yet been systematically and comprehensively elucidated.

Procyanidin C1 (PCC1), a polyphenolic component of grape seed extract (GSE), is the main substance mediating the senolytic effect of GSE. It can increase the accumulation of ROS in the cytoplasm of senescent cells and induce a continuous decrease in the mitochondrial membrane potential of senescent cells followed by the release of mitochondrial cytochrome c, resulting in the abnormal cleavage of caspase 3 and ultimately causing senescent cell apoptosis. In contrast to other traditional senolytics, PCC1 has a dual function of inhibiting SASP and targeting senescent cells (Xu et al., 2021). Its efficacy and safety in antisenescence has been demonstrated in animal studies. Moreover, the concentration of PCC1 *in vivo* varies among organs, and their local concentration may not achieve antisenescence effects in some tissue types. As phenolic compounds in grapes generally have disadvantages, such as poor water solubility and oral bioavailability. However, the use of solid lipid nanoparticle drug delivery systems to encapsulate GSE containing proanthocyanidins not only alleviates these problems but also greatly reduces oxidative stress and inflammation (Castellani et al., 2018). Astaxanthin works in the same manner as PCC1, which can ameliorate IPF through the ROS-dependent mitochondrial signaling pathway in AECIIs (Song et al., 2014b). However, the therapeutic role of PCC1 and astaxanthin in IPF should be further investigated.

Aging is an irreversible process. Senescent cells are the sources of SASP, so SASP cannot be permanently eliminated. Therefore, senomorphics need to be taken for a long period to achieve maximum intervention, and the potential risks and adverse effects of senomorphics are greater than those of senolytics (Song et al., 2020). Antisenescence agents exert a positive effect on the treatment of IPF by modulating the activities of senescent cells, as demonstrated in a mouse model (Wiley et al., 2019). However, whether its long-term application promotes the proliferation of adjacent cells, accelerate telomere shortening, and thus promote aging is unclear. Therefore, long-term preclinical trials on the efficacy of antisenescence agents for patients with IPF are needed (Lagoumtzi and Chondrogianni, 2021).

### Autophagy regulators

The mechanism of impaired autophagy in IPF is unclear. Not only it can trigger fibrosis due to inadequate mitochondrial quality control but also it can lead to abnormal NF-κB signaling through p62 accumulation (Patel et al., 2012; Hill et al., 2019; Sharma et al., 2021). Autophagy regulators include the mTOR pathway-dependent inhibitors (rapamycin, torin-1, PP242h) and the direct activators of AMPK (metformin and alginate), as well as the regulators of the acetylation pathway (spermidine and resveratrol), and also classical autophagy-inducing factors such as transcription factor EB (TFEB) (Rubinsztein et al., 2012; Menzies et al., 2017; Aman et al., 2021). It was found that metformin accelerated the regression of experimental fibrosis in an AMPK-dependent manner. Mechanistically, metformin not only promoted autophagy and reduced collagen production (Kheirollahi et al., 2019), but also prevented the production of α-SMA and ECM after TGF-β1 stimulation (Rangarajan et al., 2018). The ATM kinase inhibitor KU-60019 induces the functional recovery of an autophagic system and restores mitochondrial function and metabolic reprogramming. Autophagy modulators not only promote autophagy to prevent AEC senescence and inhibit senescence-associated inflammation but also directly reduce collagen deposition (Kang et al., 2017). Thus, the modulation of autophagic pathways holds great promise in the therapeutic exploration of IPF. The compounds of autophagic regulators are listed in Table 1.

**TABLE 1:** Autophagy regulators.

Compounds	Mechanism of action	Experimental models	References
Rapamycin	inhibit the mTOR pathway; reduce protein synthesis; promote autophagy	human; mice; yeast	Menzies et al. (2017); Rubinsztein et al. (2012); Powers et al. (2006)
Ouabain	inhibit mTOR kinases; facilitate the clearance of endogenous tau; induce autophagic lysosomes; promote cells repair	flies; human; mice	Song et al. (2019)
Fisetin	inhibit mTOR kinases; facilitate the clearance of endogenous tau *via* TFEB and Nrf2 activation; activate sirtuins	flies; mice; worms; yeast	Kim et al. (2016); Yousefzadeh et al. (2018)
Spermidine	inhibit the EP300 acetyltransferase; promote autophagy; anti-inflammation	flies; human; worms; yeast	Pietrocola et al. (2015); Freitag et al. (2022)
Resveratrol	NAD + -dependent; NF	flies, mice, worms, yeast	Morselli et al. (2011); Wang Z et al. (2021)
Metformin	mTOR-independent; act the AMPK pathway; promote autophagy	human; mice	Menzies et al. (2017)
Trehalose	mTOR-independent; activate AMPK pathway; activate TFEB; promote autophagy	human; mice; worms	Menzies et al. (2015); Sharma et al. (2018)
Urolithin A	mitophagy-dependent (pink-1, pdr-1 or dct-1); inhibit amyloid β and tau C	mice; worms	D'Amico et al. (2021); Ryu et al. (2016)
KU-60019	inhibit ATM kinase; restore mitochondrial function; promote autophagy	mice	Kang et al. (2017)
NAD^+^	mitophagy-dependent; NAD+–SIRT axis; activate sirtuins; inhibit the deacetylation of mTOR pathways and autophagy proteins	humans, mice	Lautrup et al. (2019); Lee et al. (2008)
nicotinamide riboside	mitophagy-dependent; NAD+ precursors; activate mitophagy	humans, mice	Fang et al. (2019); Mitchell et al. (2018)
lncIAPF	block autophagy by controlling the stability of the target genes EZH2, STAT1 and FOXK1	cell, human, mice	Zhang et al. (2022)

### Stem cell therapy

Mesenchymal stem cells (MSCs) have great therapeutic potential in IPF due to their powerful paracrine, anti-inflammatory, anti-apoptotic, and immunomodulatory capabilities. MSCs can inhibit AECII senescence by regulating NAMPT-mediated nicotinamide adenine dinucleotide (NAD^+^) metabolism and attenuating fibrosis, as demonstrated in animal models (Lai et al., 2022). Thus, NAD^+^ homeostasis may be responsible for the control of IPF by MSCs. Studies on animal models have found that supplementation with NAD+ precursors, such as nicotinamide riboside (NR) and nicotinamide mononucleotide (NM) can promote autophagy and have significant anti-aging effects (Verdin, 2015; Lautrup et al., 2019; Ruaro et al., 2021). According to preclinical studies on different sources of MSCs in IPF models, human Wharton jelly-derived mesenchymal stem cells may offer prospects for stem cell therapy for pulmonary fibrosis (Saleh et al., 2022).

Stem cell therapy has promising clinical applications, but high labor costs and tumorigenesis and immunological risks limit its application. Dinh et al. (2020) have proposed that the inhalation of lung spheroid cell secretome (LSC-Sec) and exosomes (LSC-Exo) is more therapeutic and has fewer side effects. Monocyte chemotactic protein 1 (MCP-1), also known as chemokine (C-C motif) ligand 2, is down-regulated in sera from animals treated with LSC-Sec and LSC-Exo. MCP-1 plays a key role in lung inflammation, and increase in its level represents poor prognosis in patients with IPF. Hence, the nebulized inhalation of LSC-Sec and LSC-Exo not only offers an opportunity for the treatment of IPF but also provides a reliable way for assessing its prognosis. However, current researches on these compounds have found many limitations in terms of dose response, route of administration, and secretome isolation. Selecting the right stem cell dose and injection method and establishing precise isolation protocols are challenges in stem cell therapy. Rejuvenating strategies for stem-cell-based therapies for aging should be explored.

## Conclusion

AECII senescence is one of the key drivers of IPF pathogenesis (Parimon et al., 2020; Yao et al., 2021). Senescent AECII-associated SADD and SASP act together as triggers and effector molecules to promote the IPF process. How senescent AECIIs act on IPF, how it alters the lung microenvironment, and how it mediates persistent and progressive fibrosis are unclear. The role of AECIIs in IPF should be explored in depth, and *in vivo* models should be conducted for the senescent AECII population.

**FIGURE 1:**
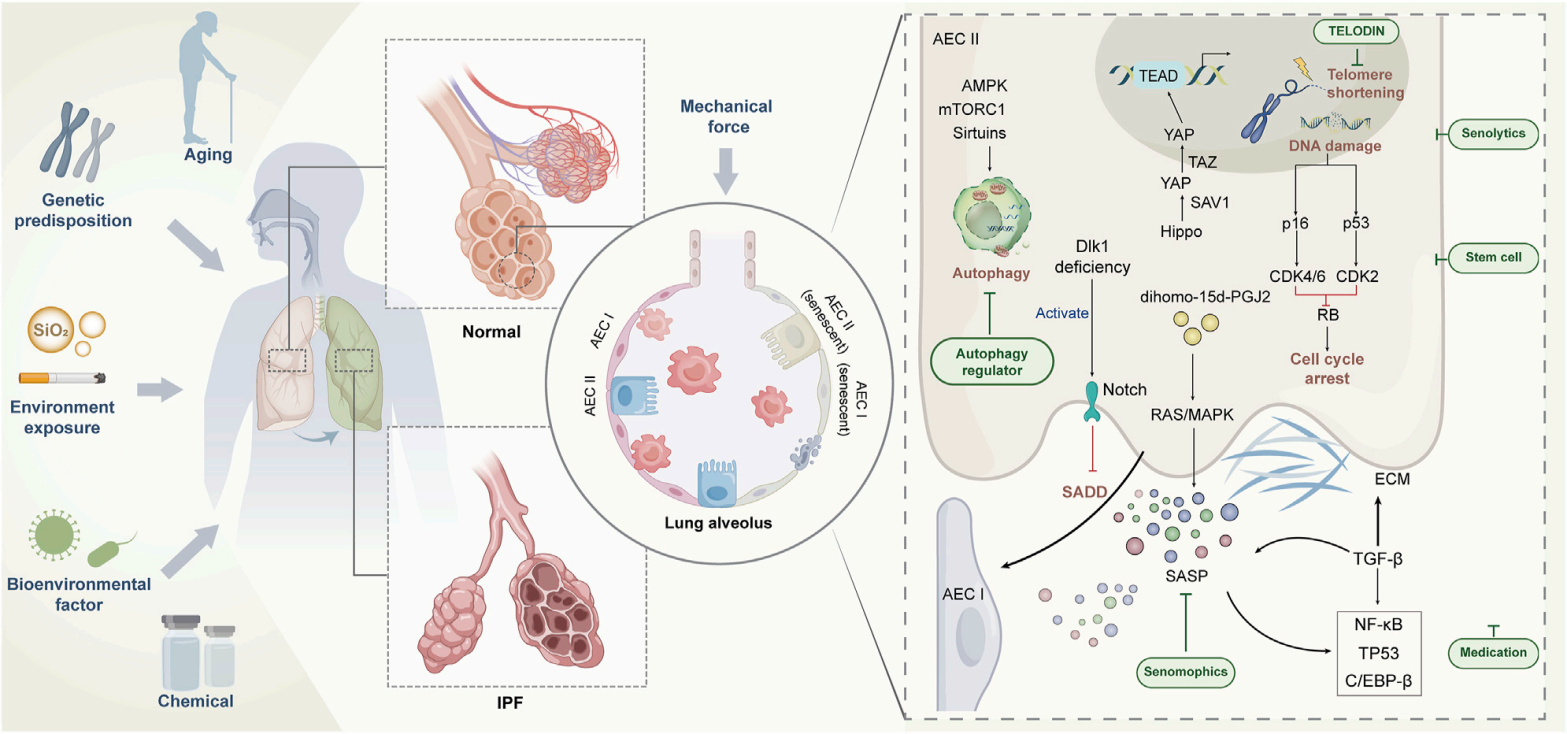
Regulation of AECII senescence and strategy of targeting senescent AECⅡ for IPF treatment.

The evolution of IPF is a dynamic process with complex etiology and unknown mechanisms. A large number of studies have revealed the complex pathophysiological basis of IPF, including genetics, epigenetics, metabolomics, and interaction with environment (Figure 1). Single-cell RNA sequencing data have shown that senescence markers are up-regulated in AECIIs in IPF (Xu et al., 2016; Mora et al., 2017; Adams et al., 2020), suggesting that impaired self-differentiation and profibrotic and low-inflammation environment caused by AECII senescence are important risk factors for IPF. Targeting senescence is expected to improve the health of patients with IPF. However, no research has confirmed that targeting senescence agents can completely remove senescent cells. However, numerous studies have shown that even partial elimination of senescent cells can cause a remission of the aging phenotype (Lehmann et al., 2017). In addition to using TELODIN and autophagy modulators in preventing AECII senescence (Hong et al., 2022), we can further investigate the selection of antisenescence agents to target senescent AECIIs or selectively block the damaging effects of SASP, thus minimizing the drive of senescence on IPF. For the acceleration of the process for targeting senescent AECIIs in the treatment of IPF, at least three issues need to be addressed: whether the progression of IPF can be terminated by targeting senescent AECIIs, whether AECII senescence can be selectively and safely targeted by some drugs without affecting normal cells, and whether clearance senescent AECIIs can reverse or even counteract the pathological features of IPF. The resolution of these issues can facilitate the design of highly effective drugs or development of novel therapies that can target senescent AECIIs and promote the restoration of the original function of senescent lung cells. In conclusion, combating or reversing lung aging by targeting senescent AECIIs is a demanding challenge. The implications of inhibiting the deterioration of IPF by medical means are objectively complex and are the subject of continued exploration in the future.
